# The rationale for detention in the Jordanian Code of criminal procedure: A comparative study with French law

**DOI:** 10.1016/j.heliyon.2022.e11164

**Published:** 2022-10-20

**Authors:** Amal Abuanzeh

**Affiliations:** University of Jordan, Queen Rania St. Amman, Jordan

**Keywords:** Detention, Presumption of innocence, Individual freedom, Jordanian code of criminal procedure, French legislation

## Abstract

Amendments to the Jordanian Code of Criminal Procedure (JCCP) concerning the rationale for detention have narrowed its scope of application and provided for alternatives. However, although these amendments are important, their drafting has not achieved the stated objectives. To address this issue, the laws of Jordan and France are reviewed. The study's novelty lies in the justification for detention in the JCCP, which takes advantage of the long experience of French law and justice. It is recommended that the Public Prosecutor and the competent court justify an extension to a period of detention or a denial of release based on Article 114(1) of the JCCP. It is also suggested that a detention warrant is only issued or extended when grounded on the details of the specific case; that is, detention must only be effected when an alternative cannot achieve one or more of the goals of detention.

## 1 Introduction

Detention is a serious restriction on an individual's liberty, as it places them in a state prison for a period determined by the competent authority, prior to a guilty or not guilty verdict for a particular crime [1]. This may conflict with the principle of presumption of innocence of the accused until proven guilty, as provided for in numerous international charters, conventions, constitutions, and national laws (see, e.g., Article 14(2) of the International Covenant on Civil and Political Rights (ICCPR) [2]; Article 6(2) of the European Convention on Human Rights (ECHR) [3]; Article 15 of the 2004 Arab Charter on Human Rights (ACHR) [4]; Article 147(1) of the Jordanian Code of Criminal Procedure [5] (JCCP); the Preliminary Article and Article 137 of the French Code of Criminal Procedure [FCCP]) [6]. Despite the gravity of detention, these international instruments and national laws recognise that it is needed so that justice can be delivered and the State's punitive policy can be implemented. In international law, detention is only allowed when strictly necessary and only as prescribed by law. Such law requires that detention is governed by strict legal provisions and guarantees to ensure a balance between the interests of the State in finding the truth and the liberty and interests of the detained individual. Article 9 of the ICCPR stipulates that, “Everyone has the right to liberty and security of person. No one may be arbitrarily arrested or detained. No one may be deprived of his liberty except for reasons provided by law and in accordance with the procedure established therein”. Article 5 of the ECHR also stipulates that detention must be exercised only by the affirmative laws of its members. Moreover, the 1953 International Conference on Penal Law stressed the importance of reasonable detention decisions [7]. To apply the provisions of this definition to the grounds or rationale for detention, the competent authority presents the legal and factual reasons leading to the instance of detention [8], wherein the latter are the evidence derived from the case [9]. In short, the rationale for detention as a principle must not only contribute to the achievement of criminal justice in the interests of all parties to the proceedings, including the complainant, the defendant, and the judiciary, it must also promote public confidence.

The grounds for detention are based on the protection of the rights of the parties to the criminal proceedings and, in particular, the right to personal freedom as guaranteed in Article 7 of the Jordanian Constitution [10]. The Constitution also states that every infringement on rights and public freedoms or the inviolability of the private life of Jordanians is a crime penalised by law. Article 8 states that:

1-No person may be seized, detained, imprisoned or the freedom thereof restricted except in accordance with the provisions of the law. 2. Every person seized, detained, imprisoned or the freedom thereof restricted should be treated in a manner that preserves human dignity; may not be tortured, in any manner, bodily or morally harmed; and may not be detained other than in the places permitted by laws; and every statement uttered by any person under any torture, harm or threat shall not be regarded.

National law also stipulates the same constitutional principle. Article 103 of the penal code provides that: "no one may be arrested or imprisoned except by order of the legally competent authorities, and also criminalises every act that constitutes an attack on individual liberty or restricts it outside the provisions of the law". Since the defendant is informed of the grounds for the denial of their liberty before being convicted, this helps strengthen confidence in judicial verdicts [11].

Transparency over the reasons for detention also helps strengthen the rights of the defence in criminal proceedings, as the detainee and their attorney can examine these reasons and how far they conform with the facts [12]; if the reasons are fictitious, the defence may prove otherwise [13]. By balancing the interests of the investigation, trial and access to the truth with those of the individual and their right to liberty before any conviction, there is a need for a compromise between the right of those detained to a fair trial and the right of society to know the truth. Knowing the grounds for detention also prevents the competent authority from abusing and abetting the rights of innocent persons [14], and guarantees the use of the power of detention within the limits established by law, knowing that there is judicial control over the execution of the conditions of detention and their practicality [15]. Transparency can also help to protect the competent authority from making wrongful and unlawful detention decisions against the defendant by building purely on the perception that detention is the best way to prove integrity and fairness. The reason for detention enhances the confidence of the general public in the impartiality of the judiciary and balances its exceptional character with the conviction of its importance, thus supporting the idea of a balance between opposing interests [16].

Considering the importance of reasonable detention, both the Jordanian and French laws have sought to state explicitly why such action may be required, in response to international requirements and in support of the presumption of innocence. The Jordanian legislature introduced an amendment to the JCCP, which explicitly stipulates the reasons for detention in Article 114(1), as follows: 1) obtaining proof of evidence; 2) preventing coercion of witnesses or victims; 3) preventing the defendant from contacting their alleged partners in a crime; 4) protecting the defendant; 5) eliminating the effect of the crime or recidivism; 6) preventing the defendant from fleeing; and, 7) sustaining public order and eliminating any disturbance [17]. Under the law of 1970, Article 144 of the French Code of Criminal Procedure provides seven reasons for detention, among others, which are generally similar to those in the Jordanian laws [18].

In Jordan, the number of persons placed in pre-trial detention was very high more than two decades ago (i.e., 47.9% of the prison population in 2005) due to lack of alternatives, traditions and routine practice to place persons arrested within the ordinary criminal justice system in detention during criminal investigation and proceedings [19]. The legislative reforms to tackle the overuse of detention began in 2001 and was followed by two subsequent amendments to the Code of Criminal Procedure (CCP) in 2009 and 2017 of which the latter entailed the most comprehensive implementation of international human rights norms including the rationale for detention. In combination, the capacity among key criminal justice institutions was supported and several pre-trial detention legal procedures were digitalized through the electronic case-management system applicable for the criminal justice system. The latest figure from 2019 showed a lower level of 36.8% of all those in detention being pre-trial detainees [20] and a decreased in the use of pre-trial detention continued to be reported in 2021 [21].

This study adopted the descriptive and comparative approach, by describing the legal texts that were organized the rationale for detention, and comparing the position of legislation, the judiciary and criminal jurisprudence in France with its Jordanian counterpart to address this issue, with reference to the position of international conventions and courts in that when the need arises. The French legal system was selected for comparison with the Jordanian since the latter is based on the former. Also, French law is experienced on the protection of individual freedoms, including control of detention and its concomitant legal guarantees, including the grounds for detention.

Simply expressing reasons for detention, however, is not a sufficient guarantee of the interests of both the State in terms of achieving justice and the individual in protecting their liberty and ensuring a fair trial. A clear mechanism is thus required so that the competent authority respects the provisions of the law and prevents arbitrary detention decisions. This mechanism differs in the Jordanian and French laws in terms of the substance and conclusiveness of the reasons for detention, the extent to which the mechanism applies to all decisions on detention, the use of detention as a last resort, and the availability of alternatives to detention. These differences are reflected in the effectiveness of the respective judiciary's control of the reasoning on detention in both countries.

In this context, the questions that arise here concern how successfully the Jordanian legislature has drafted the provisions of the amendment articles of the JCCP insofar as these state the rationale for detention, and the whether there is a need for a precise description on the rule of presumption of innocence. The study's questions are thus:

1. How do the amendments correspond to the requirements of international human rights instruments?
2. To what extent is the competent authority committed to reasonable detention?
3. Is the reasonableness of detention under the Jordanian law inclusive of all the decisions related to detention?
4. Has the Jordanian legislature successfully drafted the article amendments on alternatives to detention to ensure that detention before conviction is a last resort?
5. Do the amendments strengthen the rights of the defence and provide the detainee with sufficient safeguards to learn the reasons and necessity for their detention?

The importance of the study lies in the novelty of the justification for detention in the JCCP, taking advantage of the long experience of French law and justice in this topic, the successes and shortcomings of how the Jordanian legislature has drafted amendments to the JCCP will be addressed. Moreover, few studies have indicated a mechanism to establish reasons for detention. This study is therefore a qualitative addition which can guide judges, academics and others interested in this field, and help to establish a framework and rationale for detention decisions. The study avoids repeating Jordanian jurisprudence and the contents of the JCCP, since the amendments to its articles have not long been in effect. Noting that this study will examine the rationale for judicial detention only, not administrative, and the scope of the article is the ordinary criminal system and hence not for example the rationales of pretrial detention in the state security cases.

Instead, the aim of the study is to establish a mechanism for detention which aligns with the principle of presumption of innocence. To meet these objectives, the paper has been divided into two sections. The first covers the practical aspects of the reasons for detention and whether it is limited or widespread in Jordanian and French laws. The second focuses on when detention becomes necessary and why alternatives are inadequate.

## 2 Practical reasons for detention and its comprehensiveness in Jordanian and French law

To ensure that the reasons for detention are valid, such reasoning must be proven by facts and indicators, applicable and binding to all detention decisions, as well as proven during the criminal process. At this point, the question arises as to whether the Jordanian and French legislatures have succeeded in responding to the previously set conditions for reasonable detention and explicitly provided for detention in their respective laws.

### 2.1 Fair detention under Jordanian and French laws

In Jordanian law, before the 2017 amendment to the JCCP, no provisions were available on the justification of, and necessity for, an individual's detention. However, in 1998, the Court of Cassation made some progress towards the justification of detention, by deciding that, “Detention, the extension of the term thereof and the release of the detainee, are all conditional upon a justification. Should justification be unavailable, the decision related shall be deemed null and void” [22]. Following the 2017 amendment to the JCCP, Article 114(1) now makes explicit provision for the reasons for detention, and is regarded as a milestone in Jordanian jurisprudence as it supports a balance between individual freedoms and the interests of society. The amendment also established the notion of transparency in judicial decision-making. Each decision to detain an individual must be taken after careful examination of the legal facts of the case, thereby preventing any abuse by the competent authorities and enhancing the confidence of both the public and the parties to the criminal proceedings regarding the fairness of such decisions [23].

Considering the above, Jordanian law has become more aligned with international requirements, in particular Article 9(1) of the ICCPR. However, despite the importance of the amendment to the JCCP, there is still no explicit requirement for the Public Prosecutor to provide a reason for detention on one of the grounds set out in the preceding Article 9(1) of the ICCPR, and this failing could result in the legal text losing its binding status and weakening its control of the competent judicial authorities. Some legal provisions exist in the Jordanian Constitution and the JCCP which implicitly refer to the 2017 amendment, from which it can be concluded that detention must be effected by the competent authority. To illustrate, Articles 7–8 of the Constitution prohibit the detention of any person except in accordance with the provisions of the law, implying that the detention must be justified. Article 114 requires the Public Prosecutor to question the defendant before deciding whether to detain him or her, and so the prosecutor will have gathered sufficient information to justify either the detention or release of the defendant [24]. Article 135 of the JCCP also stipulates that decisions made by the Attorney General during the investigation stage must include the grounds for making this decision; therefore, since detention decisions are made by the Public Prosecutor, they must include the reasons for making such a decision. Considering the absence in Jordanian law of an explicit provision that obligates the causing of detention, most detention warrants are devoid of legal grounds and so many have been challenged [25].

Unlike Jordanian law, French law has established a clear, specific and explicit text for reasoning detention. The FCCP was amended in 1996 to strengthen the balance in criminal procedures by limiting the use of detention to instances of absolute necessity and providing transparency in the justification for detention [26]. Under Article 144 of the FCCP, a judge ruling on detention is obligated to justify the ruling and, in the event of any violation, a penalty of invalidity is provided for.

### 2.2 Comprehensive reasoning for detention-related decisions

To comply with Article 114(1) of the JCCP, the Jordanian legislature stipulates that the justification for detention be given only for the initiation of a detention period and not for the renewal of one, as related to the fourth paragraph of the same Article; therefore, the grounds for denial are only those in the interests of the criminal investigation. Moreover, should the release be denied, in accordance with Articles 123 and 126(1), Article 111 of the JCCP has no provision for the grounds on which the denial is based. In other words, the interest of the investigation alone is sufficient to justify the renewal of the detention, without the Public Prosecutor being obligated to base their justification on one of the seven reasons stipulated in Article 114(1) of the JCCP. This lack of an express provision to justify such decisions expands the powers of the prosecutor, so that an extension to a detention becomes automatic once the conditions for the extension required by Article 114 are applied. This neither achieves the purpose of justifying the detention nor provides sufficient guarantees for the detainee and their ability to appeal their detention, potentially making the procedure arbitrary and judicial monitoring of detention decisions difficult [27]. Clearly, these scenarios are incompatible with Article 9 of the ICCPR, which requires that all detention warrants be justified.

In contrast to the Jordanian legislature, in France, Article 137(3) of the FCCP obligates the competent judge to justify all their decisions, including decisions on detention, extensions to detention, and denials of release [28]. This obligation includes indicating the legal and factual considerations of the judicial decision and referring to one or more reasons provided for in Article 144 of the FCCP. We therefore recommend that the Jordanian legislature requires all detention decisions to be grounded in one of the reasons set forth in Article 114 of the JCCP.

### 2.3 Establishing the justification for detention

Jordanian law has no text which requires that the reasons for detention, as stipulated in Article 114 of the JCCP, be included in the detention warrant. Articles 115–116 of the JCCP stipulate the data for inclusion in the detention warrant as: the crime attributed to the accused; the legal article for which the accused is punished; the period of detention; and, the name, nickname and description of the defendant. Article 117 also stipulates that the defendant must be informed of the detention warrant but, since the reasons for the detention are not included, the accused does not know the reasons for their detention and so cannot be briefed on these grounds, to the detriment of their right to a defence. The lack of a transparent rationale also limits the exercise of judicial control over this reasoning and its legality, given that the detention warrant is subject to judicial and administrative control and there are consequences for other judicial investigation transactions [29]. In the French legislature, the system for judicial monitoring of detention warrants requires the competent judge to include the reason for detention, as provided for in Article 144, and to inform the detainee of the reason for their detention. The lack of a similar provision in Jordanian law is contrary to the requirements of Article 9(2) of the ICCPR, which requires that any detained person be informed of the reasons for their detention at the time of its occurrence.

### 2.4 Realistic grounds for detention

The Jordanian legislature implements detention on one of the grounds set forth in Article 114(1) of the JCCP. However, the Public Prosecutor is not required to justify the detention by stating the facts of the case or the indicators or criteria that strengthened their conviction that grounds for detention are available. For example, the Public Prosecutor is not obligated to explain why there is sufficient evidence to establish that a crime has occurred, and that there is a risk of flight and loss of evidence. There is also a risk that the complainant will influence witnesses or victims, or that the complainant will contact and/or collude with their partners in the crime. There is another risk that the complainant would be threatened and that there would be a risk of a breach of public order.

In practice, in Jordan the numerous decisions to implement detention have been indiscriminate and sometimes without grounds. The justification of detention is merely a formality provided for in Article 114(1), to avoid detention appeals and the fact that detention is contrary to the conditions of its adoption, without concern for the facts of the situation and the actual grounds for the prosecutor's action. Moreover, the terms used to justify detention are often cursory, such as the prevention of influence on witnesses or the protection of the defendant, and do not establish clear grounds. Instead of reflecting on the specific and factual reasons for its implementation, detention is employed in a uniform and cursory manner. Indeed, the reasons for detention provided for in Article 114 of the JCCP were flexible and broad-based in their formulation, allowing any suspect to be detained quite easily. Sometimes, these reasons are questionable, in that they may mask the primary function of detention, such as to obtain confessions, making it compelling to justify the detention of the defendant before their conviction. The court does not impose effective and genuine control over the grounds for, and practice of, detention, and nor does it not determine whether the reason for an initial detention still exists when an extension to a period of detention is made, thus failing to protect individual freedom from abuse of custody.

In contrast to the Jordanian law, Article 144 of the FCCP requires the competent judge to justify a detention order on one of the grounds provided for in the Article, and to prove that the detention is the only means available for investigation to be made, “in the light of precise and detailed elements drawn from the proceedings and the facts thereof”. The court must present the real indicators and evidence underlying its determination that a particular danger exists, and that detention is the necessary means to prevent it. This sometimes requires the judge to assess each case and ascertain the appropriateness of one of the reasons provided for in the Article. It must be borne in mind, however, that the justification for detention is difficult, as the judge must assess the possibility of future risk or dispute when selecting the appropriate reason for a detention [30]. Under French law, strict and factual justification is an important guarantee to avoid the abuse of the detention procedure [31]. In this regard, the French Court of Cassation (FCC) plays an active role in monitoring the application of this guarantee and ensuring that the reason for the detention corresponds to the facts and circumstances of the case in practice. As such, a detention order is not merely a formality to comply with the law and conditions, and is not subject to a procedural sanction. An example of how this works in practice can be seen in the FCC's dismissal of a decision by the Indictment Chamber of the Court of Appeal [32]. The latter Court had annulled an examining magistrate's decision to continue to detain a defendant on the grounds of preserving material evidence and facts, and preventing the detainee from influencing witnesses and colluding with his associates in the crime. The defendant was released on the basis that the language employed in the justification for his detention copied general and familiar language provided for in Article 144 of the FCCP, without reference to the facts and circumstances of the case. Therefore, the justification for the detention was insufficient as required under Article 145 of the FCCP.

## 3 Detention is exceptional and alternatives are inadequate

The competent authority must prove that all alternatives to detention are insufficient if the reasons for detention are to be valid. In this regard, this section deals with whether the Jordanian legislature has successfully drafted the provisions in the JCCP on this type of reasoning.

### 3.1 Detention is exceptional and unavoidable

Since detention constitutes a restriction on freedom of movement, Article 114(1) of the JCCP explicitly states that it is an “exceptional measure” only to be enforced to achieve the objectives set forth within it. In France, Article 137(3) of the FCCP iterates the principle of presumption of innocence and that the primary aim is for a suspect to remain free [33]. This therefore indicates that the rule is freedom, while detention is a last resort to achieve the ends set forth in the law. If these reasons are no longer available, the defendant must be released [34]. As such, detention orders can only be used by the Public Prosecutor as a last resort [35]. The Jordanian and French law complies with international requirements, in particular Article 9(3) of the ICCPR. Regarding this Article, the Human Rights Committee (HRC) finds that “Pre-trial detention must be the exception and must be replaced by bail, except in cases where the defendant is likely to escape, destroy evidence, influence witnesses or flee the jurisdiction of the State party” [36]. As noted by the United Nations (UN) Commission on Human Rights, detention can have a negative impact on the presumption of innocence, and thus must only be used “as a last resort in criminal proceedings, taking into account the investigation of crime and the protection of society and the victim” (UN Minimum Rules of Non-Custodial Measures, Principle 6.1). The position of the Jordanian law and French law is also consistent with the requirements of the Sixth International Conference on Penal Law, which emphasises the requirement that detention must be considered exceptional and end alongside the initial reasons for it [37]. To emphasise the exceptional nature of detention, Articles 121 and 123 of the JCCP allow the Court and the Public Prosecutor to release the accused, if this does not affect the conduct of the investigation and trial or prejudice public order; bail can be requested in return for release to ensure the presence of the accused whenever requested. This is consistent with Principle 39 of the UN Principles for the Protection of All Persons under Any Form of Detention, which provide for the “release of a detainee if detention is no longer necessary under one of the permitted grounds specified by law” [38].

### 3.2 Alternatives to detention

The exceptional nature of judicial detention is outlined in both the Jordanian and French law, both of which require that alternatives are provided for. In the Jordanian legislature, the 2017 amendment to Article 114 of the JCCP provides various alternatives to detention: “Electronic control, travel ban and house arrest or geographical area for the period specified by the Public Prosecutor or the Court, deposit of a sum of bail or provision of a guarantee of justice, prohibition of access to specific premises by the defendant”. These provisions empower the Public Prosecutor to resort to these alternatives if they are sufficient to fulfil the same interests as would detention.

The aim of alternatives to detention is to reduce the disadvantages of using detention as a deprivation of liberty and to replace it with procedures that are more effective and capable of countering crime, but less coercive and binding, as well as more protective of human rights and respectful of human dignity [39]. These alternatives also reduce the financial burden on the State through expenditure on detainees [40]. Such alternatives may also resolve several other problems derived from the negative effects of detention. First, they can prevent the integration of certain categories of accused persons (crimes of chance or emotion, or crimes committed by children) with serious criminals [41], and limit the chance of detainees becoming criminally schooled [42]. Another positive is that alternatives avoid the abuse of detention by competent authorities. The position of the Jordanian law on providing for alternatives to detention is in line with Article 9(3) of the ICCPR, which states that: “The general rule may not be detention of persons awaiting trial when they may be released, subject to trial guarantees, at any other stage of judicial proceedings, until the time of execution of the sentence” [43]. The HRC emphasised this meaning in *Michael and Brian Hill v. Spain* [44].

Detention and its necessity are also covered in other international cases. In *A.W. Mukong v. Cameroon*, the Court ruled that detention must not only be “lawful,” but also “reasonable” and “necessary” in all circumstances concerning the prevention of escape, interference with evidence, and the recurrence of crime [45]. Despite the positive amendments made to the JCCP of 2017 in adding alternatives to detention, these are unfortunately insufficient to reduce the use of detention in Jordan in certain circumstances. The drafting of the text of the Jordanian law does not obligate the use of alternatives to detention, and neither does it obligate the competent judge to prove that detention is the only means to achieve the objectives forth set in Article 114(1). This position does not align with international provisions which require that alternatives to detention are realistic and not excessively restrictive. States must ensure that there is a fully functioning and effective system for implementing alternatives to detention, according to the American Bar Association [46]. In an updated decision, the Jordanian Court of Cassation affirmed the priority of applying alternatives to detention. The Court ruled that the legislator must stipulate alternatives to detention for proper investigation procedures in an unobstructed manner. If the Public Prosecutor is convinced there are grounds for detention but does not wish to resort to it because of its serious and harsh effects on the accused, they may select alternative measures which are less severe [47]. While this decision is a remarkable step forward in improving the JCCP, it is hoped that the Jordanian legislature will amend Article 114 and obligate the use of alternatives to detention. In deciding to detain, extending a detention, or denying release, a judge is obliged to prove that alternatives to detention cannot achieve the objectives set forth in the Article 114 of the JCP, on the basis of the specific circumstances of an individual case.

Unlike the Jordanian legislature, the French legislature emphasises the need for a detailed rationale underlying detention decisions in Article 143(1). As a guardian of personal liberty, the examining magistrate in France must assess both the need for detention and the balance between the respective interests of the investigation and the individual. The same judge must also indicate, according to the circumstances of each case, whether an individual is being detained for the first time or for an extended period, or has been denied release. When making such decisions, the judge is obliged to state why alternative measures are insufficient, as provided for in Article 144 of the FCCP. The judge examines whether judicial control is sufficient to achieve the desired objectives and can resort to house arrest with electronic control where possible. In so doing, the French legislature has made detention a last resort [48].

In many of its decisions, the French Criminal Court of Cassation (FCCC) has exercised strict control over the implementation of the provisions and requirements of the FCCP. In response to the provisions of the law, the Court has upheld the decisions of the Indictment Chamber of the Court of Appeal regarding the detention of a defendant, and referred to the facts of the case to prove that detention was necessary [49]. The FCCC has also established that electronic surveillance and judicial oversight are no longer sufficient to achieve the objectives of detention provided for in Article 144 of the FCCP [50]. In this vein, the same Court has also ruled to detain a defendant in order to prevent them from influencing witnesses and obliterating evidence, sometimes while under electronic surveillance. Such decisions align with Articles 137 and 143–144 of the FCCP [51]. In another preliminary decision, the Court decided that electronic surveillance was insufficient to prevent the defendant from escaping (in this instance, from a hospital), thereby bringing the contested detention order in line with the aforementioned Articles [52].

In many court orders, the FCCC has invalidated many detention decisions, detention extensions, or denials to release from detention, because these decisions did not include detailed and specific evidence and facts from the case, proving that the alternatives to detention were insufficient [53]. A 2016 report by the Regional Observatory for Delinquency and Contexts in France shows how judges are interested in prioritising alternatives to pre-detention trial and the criteria they consider to determine these, including the questioning of the defendant before the decision is made. Among these criteria are the sex, criminal risk and age of the defendant. For example, if the defendant is female or old, they may be assigned to judicial supervision rather than detention [54].

The Jordanian and French legislatures have been very strict with those who fail to respect the terms of their conditional release by violating whichever detention alternatives have been imposed. Both Jordanian and French laws permit the judge to order *habeas corpus* against the defendant, detain them, and confiscate bail in favour of the treasury (Article 114(3), JCCP; Article 141(2), FCCP). In the case of such a violation, the competent judge is not bound by the requirement that detention be motivated by one of the reasons considered in this paper.

## 4 Conclusion

### 4.1 Results

Detention is a measure taken against the freedom of a person presumed innocent. The grounds for detention are considered a very important guarantee because they enhance the right to a defence by allowing the defendant to ascertain the legality of the decision and to challenge it if it is illegal, thus balancing the public interest with that of the individual. Grounds for detention also play an important role in promoting public confidence in criminal justice and constitute an essential guarantee against abuse by the competent authority. Under the 2017 amendment to the JCCP, the Jordanian legislature has made the reasons and motives for detention explicit. The amendment favours legislative progress in Jordan so that Jordanian law is more in alignment with international requirements for detention proceedings. However, difficulties remain regarding inaccuracies in the formulation of some of the reasons for detention, and the failure to provide a clear mechanism for its reasoning. Following this analysis of the legal provisions of the Jordanian and French law, the restrictions imposed by the French legislature on the competent judge constitute a guarantee for the defendant, because these restrictions automatically require the judge to justify that detention is the only way to achieve the desired objectives.

The Jordanian legislature has not improved the drafting of legal texts in certain areas through imposing a clear mechanism by which detention decisions can be ruled upon in the JCCP. In practice, the competent authority is not bound by any regulatory provisions and, if committed to it, is not required to provide the reasons on which the detention decision is based. The reasons for detention are not applicable to all detention decisions, and the legislature does not require that a justification for detention be included in the evidence of the detention warrant. Moreover, the Jordanian legislature has limited the application of alternatives to detention, and indeed the competent authority is not even required to establish the inadequacy of alternatives when making the detention decision. This fails to comply with the requirements of the ICCPR, which Jordan has ratified.

### 4.2 Recommendations

We suggest that the following recommendations to the JCCP be implemented by the Jordanian legislature:

1. Amending Article 114 to establish an explicit text that obliges the Public Prosecutor and the competent court to justify all decisions related to detention with one of the reasons stipulated in this Article;
2. Including, for the first time, decisions on detention and decisions to extend detention periods, by stipulation in Article 114(2), and decisions to refuse release in Article 123;
3. Amending the text of Articles 115–116 to add the grounds for detention to the data that must be included in the detention warrant, to ensure the defendant or a lawyer is aware of these reasons when notified of this warrant;
4. Amending the text of Article 114 and asking the Public Prosecutor or the competent court, when justifying the detention, to state the facts of the case or the indicators or criteria that strengthened their conviction that the grounds for detention are available;
5. Amending the text of Article 114 so that the use of alternatives to detention must be mandatory, and obligating the competent judge to prove, in light of the precise facts derived from the case, that detention is the only way to achieve its aims, as stipulated in Article 114(1), and the insufficiency of alternatives in this regard.

By implementing these recommendations, we hope that the Jordanian judiciary will exercise genuine control over the justification for, and practice of, detention, in order to modify the prevailing practices on detention decisions under the JCCP. This is achievable by seriously and fairly applying detention orders only as a last resort, as can be seen in the French judiciary which has clearly stipulated the standards on which reasons for detention are evaluated.

## Declarations

### Author contribution statement

Amal abuanzeh: Conceived and designed the experiments; Performed the experiments; Analyzed and interpreted the data; Contributed reagents, materials, analysis tools or data; Wrote the paper.

### Funding statement

This research did not receive any specific grant from funding agencies in the public, commercial, or not-for-profit sectors.

### Data availability statement

Data included in article/supp. material/referenced in article.

### Declaration of interest’s statement

The authors declare no conflict of interest.

### Additional information

No additional information is available for this paper.
